# Quantitative biology of hydrogen peroxide signaling

**DOI:** 10.1016/j.redox.2017.04.039

**Published:** 2017-05-08

**Authors:** Fernando Antunes, Paula Matos Brito

**Affiliations:** a Departamento de Química e Bioquímica and Centro de Química e Bioquímica, Faculdade de Ciências, Universidade de Lisboa, Lisboa, Portugal; b Research Institute for Medicines (iMed.ULisboa), Faculty of Pharmacy, Universidade de Lisboa, Portugal and Faculdade de Ciências da Saúde, Universidade da Beira Interior, Covilhã, Portugal

**Keywords:** Redox switches, Kinetics, Steady-state, Dynamic range, Response time, Information transmission

## Abstract

Hydrogen peroxide (H_2_O_2_) controls signaling pathways in cells by oxidative modulation of the activity of redox sensitive proteins denominated redox switches. Here, quantitative biology concepts are applied to review how H_2_O_2_ fulfills a key role in information transmission. Equations described lay the foundation of H_2_O_2_ signaling, give new insights on H_2_O_2_ signaling mechanisms, and help to learn new information from common redox signaling experiments. A key characteristic of H_2_O_2_ signaling is that the ratio between reduction and oxidation of redox switches determines the range of H_2_O_2_ concentrations to which they respond. Thus, a redox switch with low H_2_O_2_-dependent oxidability and slow reduction rate responds to the same range of H_2_O_2_ concentrations as a redox switch with high H_2_O_2_-dependent oxidability, but that is rapidly reduced. Yet, in the first case the response time is slow while in the second case is rapid. H_2_O_2_ sensing and transmission of information can be done directly or by complex mechanisms in which oxidation is relayed between proteins before oxidizing the final regulatory redox target. In spite of being a very simple molecule, H_2_O_2_ has a key role in cellular signaling, with the reliability of the information transmitted depending on the inherent chemical reactivity of redox switches, on the presence of localized H_2_O_2_ pools, and on the molecular recognition between redox switches and their partners.

## Highlights

• Hydrogen peroxide signaling proceeds through oxidation of redox switches.
• Oxidation of redox switches can be direct or mediated by highly reactive sensors.
• Response of redox switches is controlled by their oxidability and reduction rate.
• Localized protein interactions ensure the accuracy of information transmission.

## Graphical abstract

**Figure:**
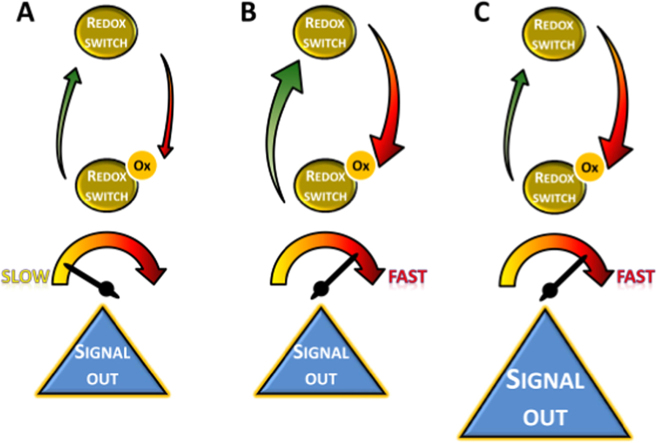

## 1 Introduction

Hydrogen peroxide (H_2_O_2_) is a non-radical oxidant present in virtually all aerobic organisms. Viewed initially as a detrimental byproduct of oxidative metabolism, today H_2_O_2_ is recognized to play important roles in cellular physiology [1]. The cellular function of H_2_O_2_ is supported by coupling of cellular signals with its production. Many enzymatic sources have been identified that produce H_2_O_2_ directly or produce superoxide radical, which is subsequently dismutated into water and H_2_O_2_, a process that is accelerated many orders of magnitude by the action of superoxide dismutases. A particularly relevant source of H_2_O_2_ is NADPH oxidases because their sole function seems to be the tightly-regulated production of superoxide/H_2_O_2_ [2].

Production of H_2_O_2_ is balanced by the action of antioxidant enzymatic systems, such as catalase, glutathione peroxidases, and peroxiredoxins, that remove H_2_O_2_ very rapidly [3], [4]. An homeostatic steady-state level of 10^−7^−10^−8^ M results under physiological conditions [5], and changes around this background steady-state level will trigger cellular responses. If these concentration shifts are moderated, transient or localized in space, being a result of for example signaling processes, a physiological stress response – or eustress – is observed [3]. If variations in the H_2_O_2_ concentration are large, sustained or affect H_2_O_2_ bulk levels, a pathological stress with deleterious effects for the organism materializes [3]. Thus, oxidative effects are inherently non-linear and biphasic with threshold levels separating the physiological and the pathological domains [6], [7]. In addition, eustress and pathological stress can either be oxidative or reductive, depending on whether they are caused by an increase or decrease of H_2_O_2_ around its background steady-state level.

In this review, quantitative biology concepts are introduced to analyze the transmission of information mediated by H_2_O_2_ in the oxidative eustress setting.

### 1.1 H_2_O_2_ signaling

Signaling pathways are regulated by the reaction of H_2_O_2_ with proteins harboring redox sensitive moieties, like metal centers or cysteine residues, whose oxidation controls their activity. These proteins denominated redox switches are key players in the regulation of biochemical pathways, including protein phosphatases, kinases or transcription factors [8]. Thus, a change in the concentration of H_2_O_2_ is matched by a change in the oxidation state of a redox switch, regulating a downstream pathway and transducing the information encoded in the H_2_O_2_ concentration profile along a signaling cascade.

Chemically, most previously identified redox-controlled switches are thiol proteins [9], [10], [11], but metal switches have also been described [12], [13]. Thiol switches are proteins with cysteine residues with low pKa that favors their proton dissociation to form a thiolate at physiological pH. Thiolates have a higher reactivity towards H_2_O_2_, but the pKa of the cysteine residue is not the only determinant of the reactivity of the thiol protein with H_2_O_2_. Rather, stabilization of the transition state between H_2_O_2_ and the cysteine residue is critical to achieve high catalytic rates with the protein environment affecting the reactivity of the cysteine group [14]. Thus, reactivity of thiol proteins towards H_2_O_2_ spans several orders of magnitude, from the low 20 M^−1^s^−1^ for some protein tyrosine phosphatases, like PTP1B and SHP-2, to the high 10^7^ M^−1^s^−1^ for peroxiredoxin 2 [15].

The chemical reactivity of redox switches is a potential mechanism underlying specific biological effects caused by different concentrations of H_2_O_2_. At low H_2_O_2_ concentrations only the most reactive switches will sense H_2_O_2_, while less reactive switches will sense H_2_O_2_ at high concentrations. As will be described below, such chemical specificity based on the oxidability of the redox switch is just one of several regulatory mechanisms in H_2_O_2_ signaling.

## 2 Quantitative analysis of H_2_O_2_ signal processing

### 2.1 The steady-state approximation

**Fig. 1:**
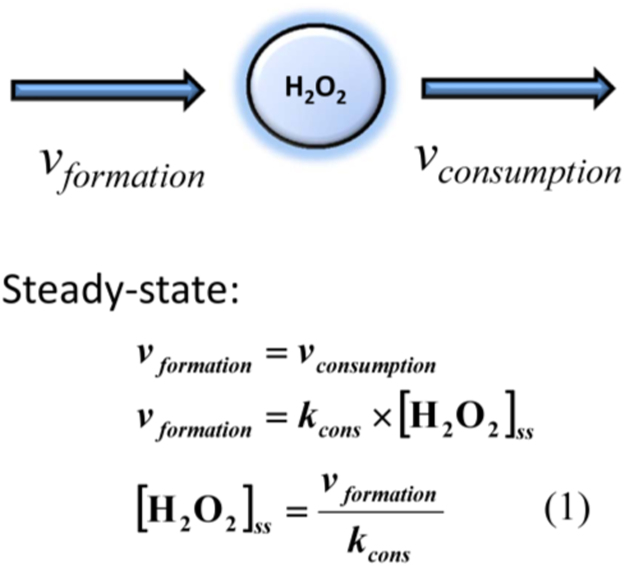
The steady-state of H_2_O_2_. A steady-state is reached when the rates of formation (*v*_*formation*_) are balanced by the rates of elimination (*v*_*consumption*_). The rate of H_2_O_2_ elimination is assumed to follow first-order kinetics because in the eustress domain H_2_O_2_ does not overload the antioxidant systems. Thus, *v*_*consumption*_= *k*_*cons*_×[H_2_O_2_] with *k*_*cons*_ being the pseudo first-order rate constant for the overall consumption of H_2_O_2_. The steady-state Eq. 1 is deduced from the equality between the rates of formation and elimination of H_2_O_2_.

**Fig. 2:**
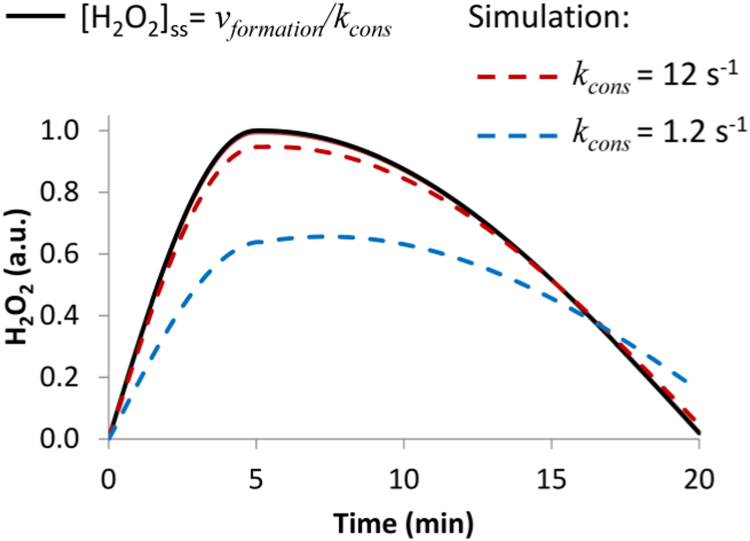
Application of the steady-state approximation to H_2_O_2_ dynamics during signaling events. To reproduce a transient H_2_O_2_ increase, the rate of H_2_O_2_ formation was assumed to peak at 5 min and to decay to zero at 20 min as observed in Ref. [19]. Three H_2_O_2_ profiles are shown: one was calculated according to steady-state Eq. 1 and two according to simulations reproducing the cell behavior for two values of consumption rate constants – 1.2 s^−1^ and 12 s^−1^. Simulated H_2_O_2_ profiles approach that calculated from Eq. 1 when the value of *k*_*cons*_ increases, and for *k*_*cons*_=120 s^−1^ or higher, simulation curves coincide with the steady-state curve (not shown). This trend is justified by the very fast time scale of the *k*_*cons*_ rate constant. A time scale of 0.06 s is calculated according to the formula *t*_*1/2*_= ln(2)/*k*_*cons*_, with ln(2) being the natural logarithm of 2, for a *k*_*cons*_ =12 s^−1^, a lower limit for the value of the *k*_*cons*_ rate constant (see Table 1 below). A *t*_*1/2*_ value of 0.06 s is much faster than the time scale associated with the variation of H_2_O_2_ formation during signaling events, which is in the minute range, and thus the steady-state approximation is valid. In general, the steady-state approximation is a reasonable assumption when analyzing processes in the minute range or slower because antioxidant systems are usually fast enough.

The steady-state concept is central to quantitative analyses in redox biology [16], [17], [18]. As a result of continuous formation and elimination, H_2_O_2_ settles to a near steady-state given by Eq. 1 in Fig. 1. It is important to test the validity of the steady-state approximation during signaling events when variations of the H_2_O_2_ concentration are observed. In other words, does the steady-state approximation hold when H_2_O_2_ is not steady? When H_2_O_2_ production is increased, for example due to the activation of an NADPH oxidase, the steady-state approximation can be used to calculate the transient dynamics of H_2_O_2_ because the very fast elimination of H_2_O_2_ by antioxidants systems has a reaction time much quicker than the transient responses formed during signaling events (Fig. 2). Thus, the steady-state approximation is valid even when H_2_O_2_ levels change during signaling events.

**Fig. 3:**
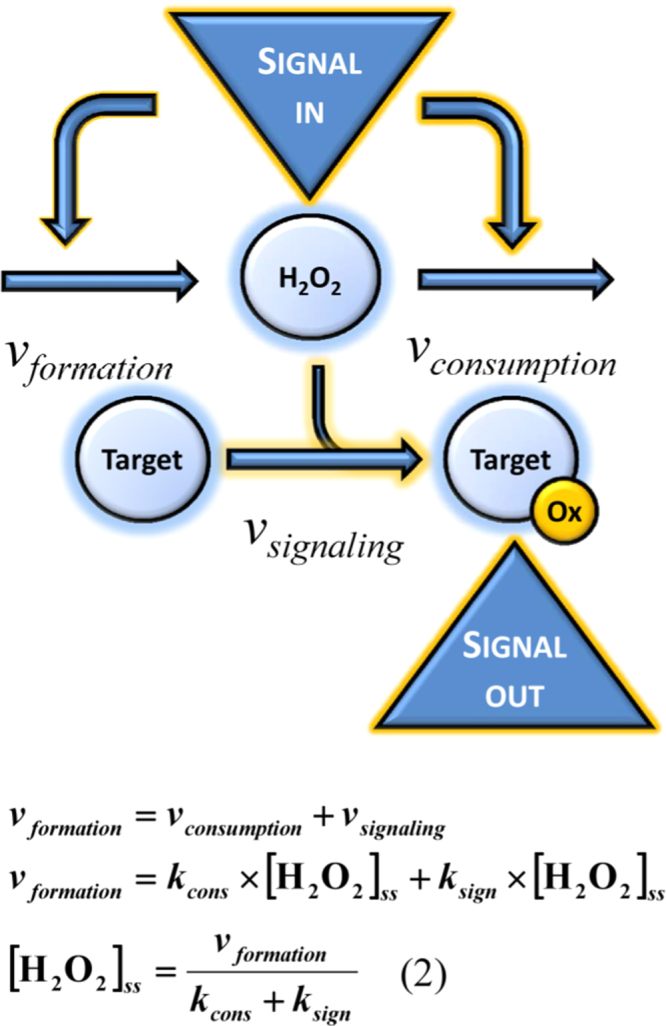
The H_2_O_2_ steady-state in the presence of signaling. In principle, a signal (Signal in) can modulate either the production or the removal of H_2_O_2_, the activation of a NADPH oxidase being a common mechanism. The subsequent change in H_2_O_2_ concentration is sensed by a redox switch (Target) that upon oxidation (*v*_*signaling*_) transmits information downstream the signaling cascade (Signal out). Similarly to the rate of H_2_O_2_ consumption by antioxidant systems, the signaling reaction also follows first-order kinetics, being *v*_*signaling*_=*k*_*sign*_×[H_2_O_2_] with *k*_*sign*_ referring to the rate constant for the reaction of H_2_O_2_ with the redox target. The resulting steady-state H_2_O_2_ concentration is given by Eq. 2.

To make a quantitative analysis of H_2_O_2_ signal processing, the simple steady-state scheme of Fig. 1 was extended to include a signaling reaction. The formation of H_2_O_2_ is now balanced by two elimination reactions, one being the consumption of H_2_O_2_ by antioxidant systems and the other the oxidation of redox switches (Fig. 3). When first-order kinetics are assumed for these elimination processes, H_2_O_2_ steady-state is given by Eq. 2 in Fig. 3.

Eq. 2 shows the relative magnitude of *k*_*cons*_ and *k*_*sign*_ only is needed to predict whether signaling processes affect directly the H_2_O_2_ steady-state. According to published data, *k*_*cons*_ is five to six orders of magnitude higher than *k*_*sign*_ (Table 1), implying that antioxidant reactions vastly outcompete signaling reactions for H_2_O_2_. Thus, a kinetic bottleneck for H_2_O_2_ signaling is established [10], [14], [15], [20]. If highly efficient antioxidant systems divert more than 99.999% of H_2_O_2_ from signaling reactions, how are H_2_O_2_ variations sensed? The rate of signaling is calculated as the product of the rate constant *k*_*sign*_ by the concentration of H_2_O_2_ (Fig. 3). So, the rate of the signaling reaction will match the variations of H_2_O_2_, and the information encoded in the H_2_O_2_ concentration profile can, in principle, be transmitted downstream the signaling cascade. The key question is whether the information is transferred fast enough when *v*_*signaling*_ is very slow.

**Table 1:** Competition between enzymatic antioxidants and redox switches for H_2_O_2_. *k*_*cons*_ and *k*_*sign*_ are pseudo first-order rate constants calculated as the product between *k*_target+H2O2_, the chemical rate constant between H_2_O_2_ and its target protein, and the concentration of the target protein, either an enzymatic antioxidant or a redox switch. For protein concentrations and rate constant values see [15], [8]. The rather high value for catalase concentration refers to the peroxisome [21].

	***k***_***target+H2O2***_ ***(M***^***−1***^***s***^***−1***^***)***	***[protein]*** ***(µM)***	***k***_***target+H2O2***_***× protein (s***^***−1***^***)***
**Antioxidant**			*k*_*cons*_
*Prx*	10^5^–10^7^	10	1–100
*GPx*	6×10^7^	0.2–10	12–600
*Catalase*	10^7^	10^3^	10^4^
**Redox switch**			*k*_*sign*_
*PTP1B*	24	0.01	2.4×10^−7^
*SHP‐2*	20	0.01	2.0×10^−7^
*Keap1*	140	1	1.4×10^−4^

Prx – Peroxiredoxin; GPx – Glutathione peroxidase 1.

### 2.2 Equations governing H_2_O_2_ signaling

**Fig. 4:**
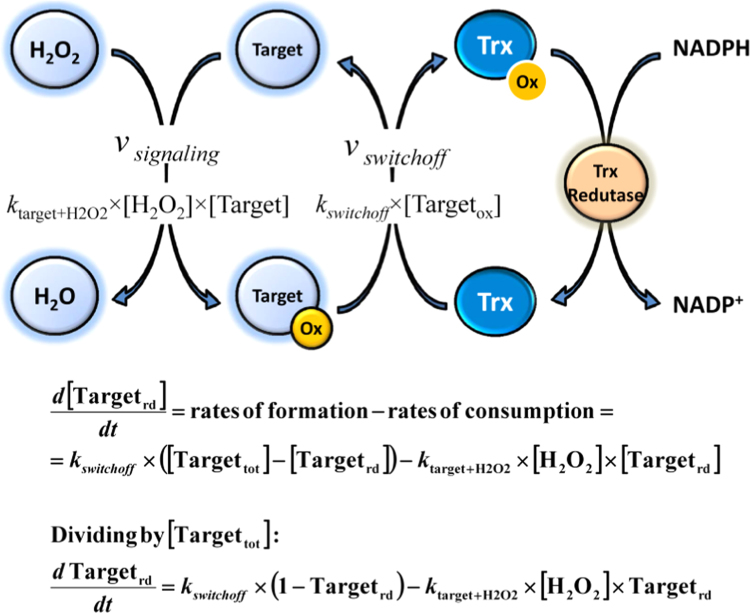
A minimal model of H_2_O_2_ signaling mediated by redox switches. A redox switch (Target) is oxidized by H_2_O_2_ and then reduced by, for example, a member of the thioredoxin family (Trx). The rate of target oxidation is equaled to the rate of the chemical reaction between H_2_O_2_ and the reduced form of the target (*k*_target+H2O2_×[H_2_O_2_]×[Target_rd_]), and the rate of reduction is set to *k*_*switchoff*_×[Target_ox_], with *k*_*switchoff*_ being a pseudo first-order rate constant. The differential equation is built, simplified by dividing by the total concentration of target [Target_tot_], and solved with a software like Maxima [24]. The resulting solution describes the time course of the target in terms of its molar fraction in the reduced state (Target_rd_), thus avoiding the utilization of absolute concentrations, a measure that is difficult to measure experimentally.

**Fig. 5:**
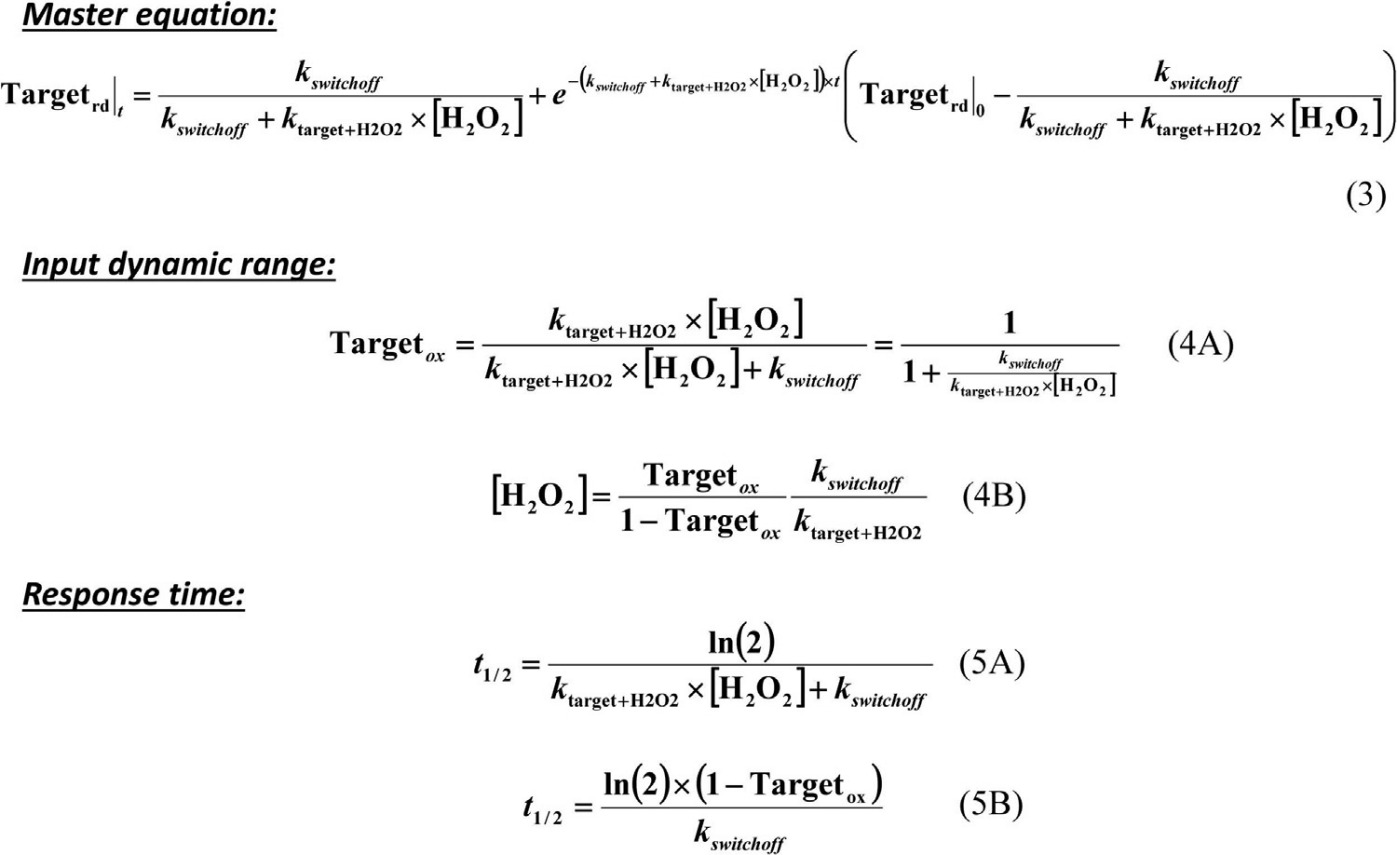
Governing equations of H_2_O_2_ signaling. The master equation (Eq. 3) includes the dependence on the sustained H_2_O_2_ signaling concentration [H_2_O_2_] attained in the vicinity of the redox switch during the signaling process, as well as the rate constants for oxidation (*k*_target+H2O2_) and reduction (*k*_*switchoff*_) of the redox switch, and the fraction of the redox switch in the reduced form at time 0 (${{\mathrm{Target}}_{\mathrm{rd}}|}_{0}$). The key features of H_2_O_2_ signaling are described by two sets of simpler equations deduced from the master Eq. 3 [8], [23]. Eq. 4A is deduced by letting *t* tend to infinite and represents the steady-state fraction of the redox switch in the oxidized form (Target_ox_), which is a measure of the amount of information transmitted from H_2_O_2_ to the redox target. Calculation of the H_2_O_2_ signaling concentration causing a certain steady-state value of target oxidation is done with Eq. 4B, which results from an arrangement of Eq. 4A. The second set of equations (Eqs. 5A and 5B) calculates the response time of the redox switch to H_2_O_2_, giving the time (*t*_*1/2*_) needed for oxidizing half of the target present initially, i.e., indicating whether transmission of information proceeds rapid enough. Eq. 5A is deduced by replacing ${{\mathrm{Target}}_{\mathrm{rd}}|}_{t}$ by ${{\mathrm{Target}}_{\mathrm{rd}}|}_{0}/2$ and *t* by *t*_*1/*2_ in Eq. 3 and calculates *t*_*1/2*_ as a function of H_2_O_2_ concentration. Eq. 5B is deduced by replacing Eq. 4B in Eq. 5A and calculates *t*_*1/2*_ as a function of the steady-state fraction of the redox switch in the oxidized form and on the value of *k*_*switchoff*_.

The issue whether a slow chemical reaction between H_2_O_2_ and a redox switch ensures timely information transmission during signal processing may be addressed with the help of the minimal mathematical model shown in Fig. 4. This model is formed by the oxidation-reduction cycle of a redox switch, which may be viewed as a switch-on switch-off sequence [22] with the on state – oxidized form of the redox switch – relaying the information encoded in H_2_O_2_ down the signaling cascade. The solution of this model yields a master equation (Eq. 3 in Fig. 5) describing the time course of the fraction of the redox switch in the reduced form [23]. From Eq. 3 two sets of simpler equations are deduced (Fig. 5), namely (i) Eqs. 4 A and 4B describing the effect of input H_2_O_2_ concentrations on the signaling response, and (ii) Eqs. 5 A and 5B characterizing the time-dependent H_2_O_2_ signaling properties. The input H_2_O_2_ concentrations and the response time are two important quantitative measures of redox signaling proposed before [18].

### 2.3 H_2_O_2_ dynamic range

**Fig. 6:**
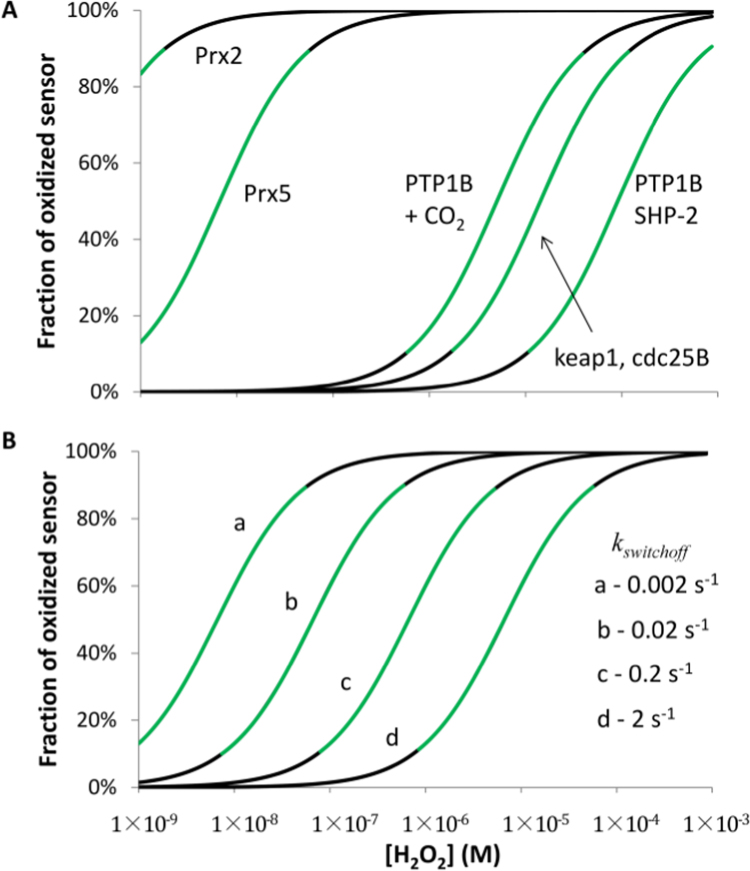
H_2_O_2_ input dynamic range for redox switches. Plots of Eq. 4A show the range of H_2_O_2_ concentrations to which thiol proteins with different reactivity respond. The following *k*_target+H2O2_ values were used: peroxiredoxin 2 (Prx2) – 1×10^7^ M^−1^s^−1^; peroxiredoxin 5 (Prx5) – 3×10^5^ M^−1^s^−1^; Kelch-like ECH-associated protein 1 (Keap1) – 140 M^−1^s^−1^; cell division cycle 25B (cdc25B) – 140 M^−1^s^−1^; protein tyrosine phosphatase 1B (PTP1B) – 24 M^−1^s^−1^; PTP1B in presence of bicarbonate (PTP1B + CO_2_) – 396 M^−1^s^−1^; and, *src*-homology 2 containing tyrosine phosphatase (SHP-2) – 20 M^−1^s^−1^. In (A) *k*_*switchoff*_ value was 2×10^−3^ s^−1^, while in (B) the influence of a range of *k*_*switchoff*_ values is shown for peroxiredoxin 5. Input H_2_O_2_ concentrations sustaining information transmission by redox switches are shown in green and were defined as the input eliciting a 10–90% oxidation of the protein. Below 10% oxidation, redox switch response is considered too weak to transmit efficiently the H_2_O_2_ signal, while above 90% oxidation, the response is near saturated to further increase in the H_2_O_2_ concentration.

The input dynamic range, i.e. the range of H_2_O_2_ concentrations for which redox switches act as sensors of H_2_O_2_, depends on the reactivity of the redox switch towards H_2_O_2_, being inversely proportional to *k*_target+H2O2_ (Fig. 6A). One source of uncertainty in the plot of Fig. 6A is the *k*_target+H2O2_ value because, among other factors, H_2_O_2_ reactivity increases several orders of magnitude upon reaction with phosphate and carbon dioxide yielding peroxymonophosphate [25] and peroxymonocarbonate [26], [27], respectively. The reactivity of PTP1B with peroxymonophosphate is 7000-fold higher than with H_2_O_2_ itself [25], and at pH 7, the presence of carbonate at 25 mM, a physiological level, accelerates the reaction between PTP1B and H_2_O_2_ from 24 M^−1^s^−1^ to 202 M^−1^s^−1^ at 25 °C and to 396 M^−1^s^−1^ at 37 °C. The formation of these derivatives is not immediate, taking 5–8 min to reach an equilibrium with H_2_O_2_ [25], [27]. Nevertheless, for peroxymonocarbonate, PTP1B accelerates this equilibration to a few seconds or faster [26]. This effect was attributed to oxidation of the active-site cysteine by peroxymonocarbonate possibly formed in the active center of the enzyme [26]. Zn(II) complexes and other Lewis acids increase the rate of peroxymonocarbonate formation [27], and one may speculate that Arg221, being present in the active site of PTP1B and being essential for catalysis [28], can act as a Lewis acid catalyzing the formation of peroxymonocarbonate. Therefore, in Fig. 6A the input dynamic range was also calculated for PTP1B in the presence of CO_2_.

In addition to the reactivity of the redox protein towards H_2_O_2_, the input dynamic range also depends on the rate of reduction of the redox switch, increasing for high *k*_*switchoff*_ rate constants, as shown in Fig. 6B. Thus, according to Eqs. 4A and 4B the reduction of the redox switch inhibits the transmission of H_2_O_2_ signals, as it is observed for the reductions of OxyR by glutaredoxin 1 [29], of Yap1 by thioredoxins 1 and 2 [30], of Pap1 by thioredoxins 1 and 3 [31], [32], of PTP1B by redoxin TRP14 and thioredoxin 1 [33], [34], of PTEN by thioredoxin 1 [34], and of the NRF2/KEAP1 system by the thioredoxin system [35].

*k*_*switchoff*_ values for protein phosphatases PTP1B and SHP-2 – 2×10^−3^ s^−1^ – [23] are about three orders of magnitude lower than for peroxiredoxins – 2 s^−1^ [36]. This large difference reflects the value of the rate constant for the reduction of phosphatases – 700 M^−1^s^−1^ for PTP1B (estimated from [37]) – being much lower than the rate constant for the reduction of peroxiredoxins by thioredoxin – 2.1×10^5^ M^−1^s^−1^ [38], 2.2×10^5^ M^−1^s^−1^ (estimated from [39]) and (2–8)×10^5^ M^−1^s^−1^ (estimated from [40]) for peroxiredoxins 2, 3, and 5, respectively. Thus, the input dynamic range for peroxiredoxins is not as low as it could be expected from their high reactivity towards H_2_O_2_, and may even overlap with that of less reactive proteins. For example, input dynamic ranges for PTP1B and peroxiredoxin 5 are predicted to overlap (Table 2).

**Table 2:** H_2_O_2_ dynamic range and response time for thiol proteins. Calculation of H_2_O_2_ dynamic range was done with Eq. 4B, assuming 10% and 90% of target oxidation, respectively for the lower and upper limit. Calculation of the response time to H_2_O_2_ was done with Eq. 5B, assuming 50% of target oxidation. For PTP1B and SHP-2 data for the reactivity obtained in the presence of CO_2_[26] is also shown.

***Redox target***	***k***_***target+H2O2***_***(M***^***−1***^***s***^***−1***^***)***	***k***_***switchoff***_***(s***^***−1***^***)***	***H***_***2***_***O***_***2***_***dynamic range (µM)***	***Response time to H***_***2***_***O***_***2***_***(s)***
*PTP1B*	24	2×10^−3^	9–750	173
*+CO*_*2*_	396	2×10^−3^	0.6–45	173
*SHP‐2*	20	2×10^−3^	11–900	173
*+CO*_*2*_	167	2×10^−3^	1.3–108	173
*Prx5*	3×10^5^	2	0.7–60	0.2
*Prx2*	1×10^7^	2	0.02–1.8	0.2

Table 2 shows the input dynamic range predicted according to published kinetic data. For H_2_O_2_ signaling concentrations lower than 1 µM, signaling is probably intermediated by a high reactive protein such as peroxiredoxin 2. For H_2_O_2_ concentrations higher than 1 µM, mediation of signaling by proteins with different reactivity towards H_2_O_2_ is feasible, but mediation by a high or by a low reactive protein is not equivalent as the response time is different (see below).

Not considered here is the hyperoxidation of peroxiredoxins, which also mediates transmission of information encoded in H_2_O_2_ [41], [42], [43]. Having different input dynamic ranges depending on modified forms of the same protein gives adaptability to signaling systems [44].

### 2.4 Response time to H_2_O_2_

Similarly to the input range, the response time also depends on the reactivity of proteins towards H_2_O_2_ and on the value of *k*_*switchoff*_ (Eqs. 5A and 5B). But contrary to the input dynamic range, the response does not depend on the ratio of the rate constant values for these processes, but rather on their sum. Therefore, between two proteins with similar input dynamic ranges, the one with higher reactivity towards H_2_O_2_ displays a faster response time for a given H_2_O_2_ concentration. For example, PTP1B will respond with a time of approximately 3 min, while peroxiredoxin 5 responds in less than 1 s, even if they have similar input dynamic ranges (Table 2). The response time for PTP1B calculated here is much faster than published estimations [8], [26], [36] because previous analyses considered only the oxidation of the redox switch, neglecting the impact of its reduction in the acceleration of the response time.

**Fig. 7:**
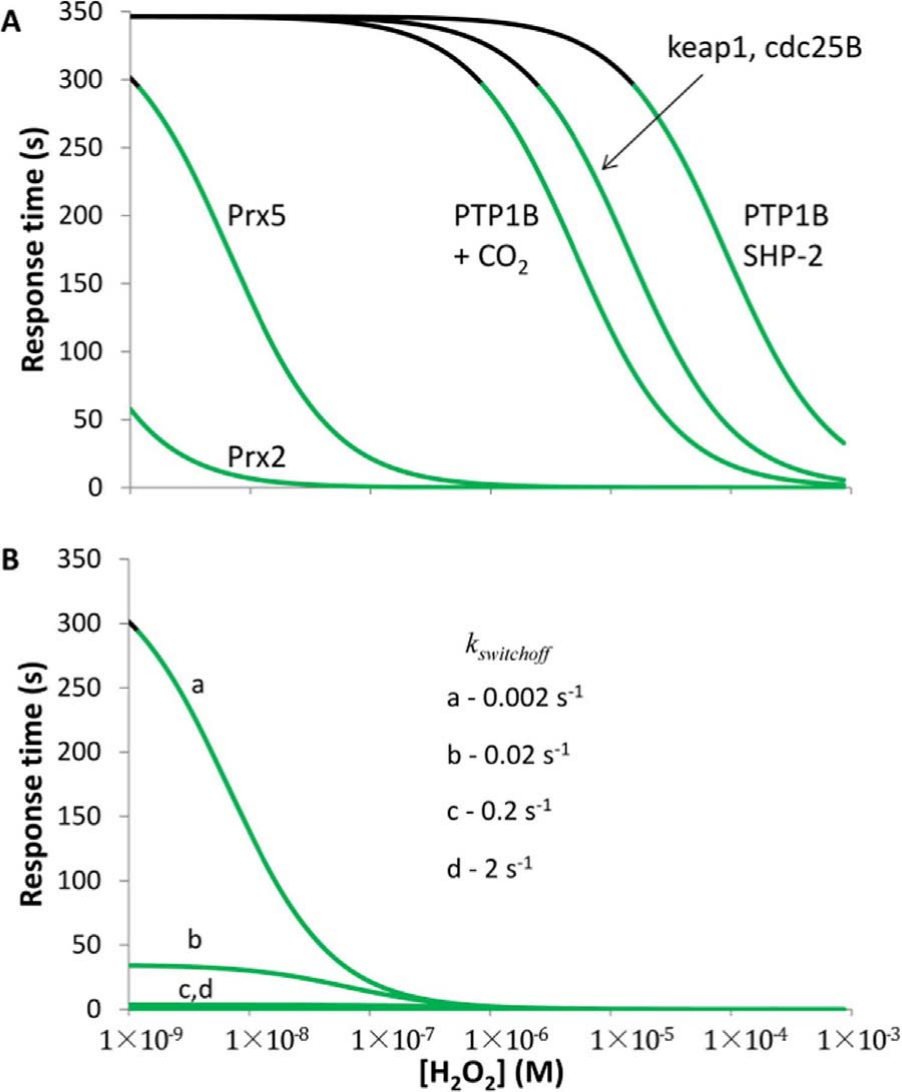
Response time of redox switches to H_2_O_2_. Plots of Eq. 5A show the time needed to reach 50% of the overall response of sensors to H_2_O_2_. Values of rate constants are the same as those used in Fig. 6. In (A) *k*_*switchoff*_ value was 2×10^−3^ s^−1^, while in (B) the influence of a range of *k*_*switchoff*_ values is shown for peroxiredoxin 5. Input H_2_O_2_ concentrations supporting rapid information transmission by redox switches are shown in green and were defined as the input eliciting responses faster than 10 min. Nonetheless, some processes may be compatible with longer responses times to H_2_O_2_, including for example apoptosis [45] or adaptation pathways [46], [47].

Response time values shown in Table 2 were calculated with Eq. 5 B, which hides the influence of H_2_O_2_ concentration and *k*_target+H2O2_. For example, in spite of very different reactivity towards H_2_O_2_, peroxiredoxins 2 and 5 have a similar response time – 0.2 s. Implicit H_2_O_2_ concentrations used are, however, different for peroxiredoxins 2 and 5, being those that induce 50% of peroxiredoxin oxidation, as calculated by Eq. 4B. For the same H_2_O_2_ concentration, peroxiredoxin 2 has a much faster response time than peroxiredoxin 5, as shown in Fig. 7A.

### 2.5 Analyzing typical experiments

In addition to provide new insights on the mechanisms of H_2_O_2_ signaling, the two sets of Eqs. 4 and 5 may be applied to learn new information from typical redox signaling experiments that measure the oxidation time course of redox switches. To this end, three experimental measurements are useful: (i) the fraction of the redox target in the oxidized form, (ii) the response time, and (iii) the H_2_O_2_ input dynamic range. The oxidation levels of the redox target and the response time can be estimated from the time course of the oxidation profile of the target under analysis. The measurement of the input dynamic range is more difficult. When H_2_O_2_ is added externally, the intracellular concentration of H_2_O_2_ is lower than that applied extracellularly, and a gradient across the plasma membrane is established [48]. The magnitude of this gradient is unknown and depends on the cell type and whether peroxiredoxins are active at the external H_2_O_2_ concentration applied in the experimental set up. The presence of active peroxiredoxins increases gradients by approximately two orders of magnitude, from values under 10 [48], [49], [50] to values in the 650–1000 range [51], [52]. Thus, uncertainties in the values of intracellular H_2_O_2_ concentrations impact the determination of the input dynamic range when H_2_O_2_ is added extracellularly. Alternatively, if endogenous production of H_2_O_2_ is stimulated with a signaling molecule, like a growth factor, the intracellular H_2_O_2_ concentration is also unknown. Even if the intracellular H_2_O_2_ level is followed with a probe, the conversion of the signal measured to H_2_O_2_ concentrations values is usually not done. In spite of these caveats, useful information can still be obtained from the concentration of H_2_O_2_ applied in experiments as exemplified below.

Stat3 is inhibited by 5 µM extracellular H_2_O_2_ [53], which corresponds to an intracellular concentration in the range of 0.5–0.005 µM if gradients are considered. In spite of this wide range of possible H_2_O_2_ concentrations, the involvement of a sensor with reactivity similar to peroxiredoxin 2 can be predicted (Table 2). In addition, the response time observed experimentally is below 60 s, for an extracellular H_2_O_2_ concentration of 50 µM [53]. A peroxiredoxin-like sensor is needed to attain such rapid response according to Eqs. 5 A and 5B. In fact, peroxiredoxin 2 acts as a sensor, reacting with H_2_O_2_ and then relaying the oxidation to form disulfide links between Stat3 monomers [53]. Of note, when oxidation relays are involved response times are slower than those indicated in Table 2 as the oxidation relay step introduces an additional delay not considered in the minimal model of Fig. 4.

In another example, a response time of about 4–5 min is estimated from the PTP1B oxidation profile observed when the endogenous production of H_2_O_2_ is triggered by EGF in A431 cells [54]. This slow response time is compatible with the direct reaction of H_2_O_2_ with PTP1B (see Table 2). In addition, in this case the H_2_O_2_ signaling concentration attained in the vicinity of PTP1B can be estimated as rate constants for PTP1B are known: if the level of PTP1B oxidation measured experimentally – approximately 50% [54] – together with *k*_target+H2O2_ =396 M^−1^s^−1^ and *k*_*switchoff*_ =2×10^−3^ s^−1^, is introduced in equation 4B, a concentration of 5 µM is estimated. By a similar approach, the H_2_O_2_ concentration attained in the vicinity of SHP-2 during stimulation of Rat-1 cells by PDGF is calculated to be 6 µM, based on the observed SHP-2 oxidation profile [55]. H_2_O_2_ concentrations in the order of 5–6 µM are much higher than the bulk steady-state H_2_O_2_ concentration, estimated in the range 0.1–0.01 µM [5], but are still plausible as localized pools of H_2_O_2_ probably play an important role during signaling [13], [36], [41], [56]. The plausibility of this estimation is reinforced by noting that oxidation profiles of protein phosphatases PTP1B and SHP-2 observed with growth factors are similar to those observed with extracellular H_2_O_2_ concentrations close to 100 µM [23], [55], [57]. An extracellular 100 µM H_2_O_2_ concentration corresponds to an intracellular concentration of 5 µM if an H_2_O_2_ gradient across the plasma membrane of 20 is established, which is plausible taking into consideration that at this relatively high external H_2_O_2_ levels peroxiredoxin systems are at least partially inhibited [36], [58].

Nonetheless, H_2_O_2_ concentrations attained during signaling are most probably pathway dependent. As referred above, low H_2_O_2_ extracellular concentrations, in the order of 5 µM, are in play during the formation of disulfide-linked Stat3 oligomers [53]. On the other hand, the inhibition of protein phosphatase 1 (PP1) probably needs a high dose of H_2_O_2_ because an associated IC_50_ of 67 µM was measured in vitro [13]; in fact, this inhibition is probably mediated by localized production of H_2_O_2_ because colocalization of NOX4 and PP1 was observed and both proteins were identified together in a complex [13].

The previous discussion illustrates how Eqs. 4 and 5 give new insights on the mechanisms of H_2_O_2_ signaling and how new information is learned from common experimental measures. In addition, other applications for the equations are possible, including for example their fitting to experimental data to determine rate constants [23].

## 3 Final remarks

**Fig. 8:**
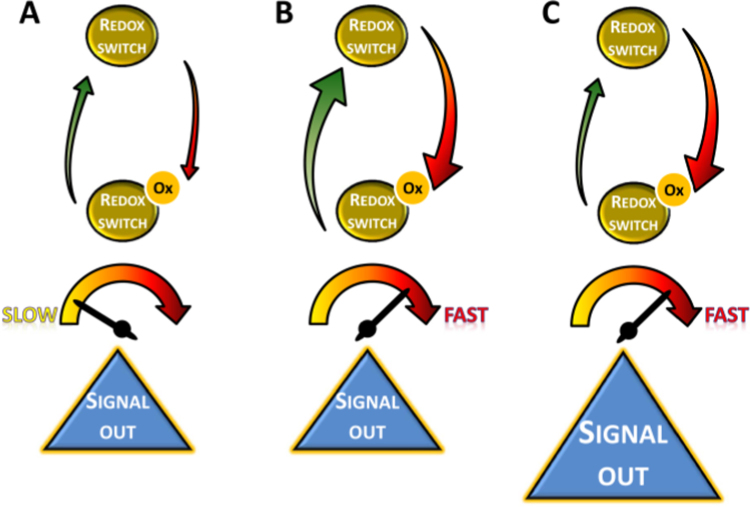
Redox signaling properties. Redox characteristics of the redox switch define the properties of the transmission of information encoded in H_2_O_2_. When the ratio between oxidation and reduction is similar the same information output is observed (panels A and B), but the redox switch with higher redox turnover has a faster response (panel B). If oxidation dominates over reduction, signal transduction is favored by displacing the redox switch to the oxidized form (Panel C).

The main results of the quantitative biology analysis of H_2_O_2_ signaling presented here are summarized in the form of Eqs. 4 and 5 and are depicted in Fig. 8. Eqs. 4 and 5 govern the biology of H_2_O_2_ signaling and provide a quantitative framework with predictive power. In addition, common experimental measurements, like response time and oxidation profile of redox switches, may be analyzed with these equations, giving hints on the mechanisms of signal processing underlying experimental observations. Although the analysis was focused on thiol switches, these equations also apply to other types of redox switches.

The input dynamic range, i.e., the H_2_O_2_ concentration range to which redox switches respond, depends not only on their H_2_O_2_-induced oxidability but also on their rate of reduction (Fig. 8). Eq. 4 indicates that the ratio between the kinetics constants of these two processes defines the sensitivity of the redox switch to H_2_O_2_. Peroxiredoxins and protein phosphatases respond in ranges of H_2_O_2_ concentration that are not as far apart as it could be predicted based solely in their very large different reactivity towards H_2_O_2_, because peroxiredoxins are reduced faster than protein phosphatases (Table 2). The response time also depends on the rates of oxidation and reduction of the redox switch given by Eq. 5. In this case, it is not the ratio between oxidation and reduction that determines the response time, but their effects add up to increase the rate of response.

**Fig. 9:**
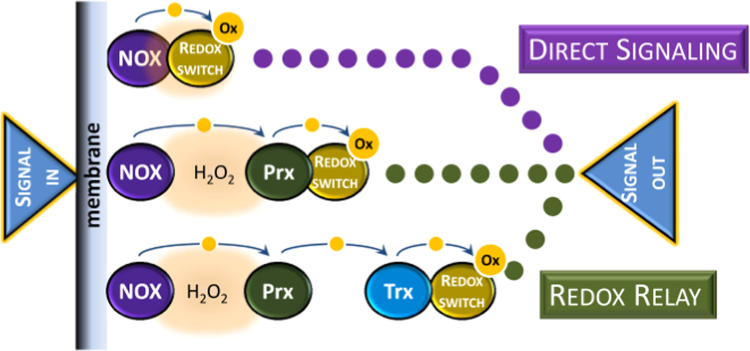
Mechanisms of H_2_O_2_ signaling. In the direct signaling pathway, H_2_O_2_ reacts directly with a redox switch. In the redox relay mechanism, the sensor of H_2_O_2_, a high-reactive protein such as a peroxiredoxin, relays the oxidation to a redox switch. The redox relay mechanism can be made more complex by the intervention of additional intermediates, as exemplified in the thioredoxin-peroxiredoxin model in which the protein responsible for the reduction of the redox sensor acts as a redox relay.

Redox switches transmit information along a signaling cascade after being oxidized by H_2_O_2_. This oxidation may be direct or, alternatively, indirect when the redox sensor is a high reactive protein, like a peroxiredoxin, that relays the oxidation to a redox switch with low reactivity towards H_2_O_2_ (Fig. 9) [4], [59], [60]. Examples of relay circuits already identified include the original discovery of the Gpx3/Yap1 [61], and subsequently Tpx1/Pap1 [32], [62], Tsa1/Sty1 [63], Prx1/Ask1 [64], and Prx2/Stat3 [53]. In the thioredoxin-peroxiredoxin model, thioredoxin, or another protein responsible for the reduction of the redox sensor, acts as a redox relay, mediating the oxidation of a downstream redox switch [60], [65].

Independently of the specific mechanism, localized interactions are probably important to attain accurate information transmission [66]. These localized interactions include (1) complexes of NOX with redox switches, favoring switch on of a specific redox sensor, and (2) the interaction of a high reactive sensor, such as a peroxiredoxin, with a target protein, sustaining a specific relay of the oxidative signal. In this second case, the peroxiredoxin will not only relay the oxidative signal downstream but also trap H_2_O_2_ [36], avoiding H_2_O_2_ diffusion outside the signal locus and, consequently, preventing either unspecific signaling messages or even some form of pathological stress.

In conclusion, the relatively weak oxidation potential of H_2_O_2_ is coupled to timely and accurate transmission of information by a combination of chemical reactions that balance oxidation and reduction of redox switches, together with specific protein interactions and localized H_2_O_2_ pools.
